# Incidence, aetiology and temporal trend of bloodstream infections in southern Sweden from 2006 to 2019: a population-based study

**DOI:** 10.2807/1560-7917.ES.2023.28.10.2200519

**Published:** 2023-03-09

**Authors:** Oskar Ljungquist, Adam Blomstergren, Adam Merkel, Torgny Sunnerhagen, Karin Holm, Gustav Torisson

**Affiliations:** 1 Department of Infectious Diseases, Helsingborg hospital, Helsingborg, Sweden; 2 Division of Infection Medicine, Department of Clinical Sciences, Lund University, Lund, Sweden; 3 Clinical Infection Medicine, Department of Translational Medicine, Faculty of Medicine, Lund University, Malmö, Sweden; 4 Department of Infectious Diseases, Skåne University hospital, Malmö, Sweden; 5 Clinical Microbiology, Infection Prevention and Control, Office for Medical Services, Region Skåne, Lund, Sweden

**Keywords:** antimicrobial resistance, blood stream infections, incidence, bacterial infections, preventive medicine

## Abstract

**Background:**

Bloodstream infections (BSI) are a public health concern, and infections caused by resistant bacteria further increase the overall BSI burden on healthcare.

**Aim:**

To provide a population-based estimate of BSI incidence and relate this to the forthcoming demographic ageing western population change.

**Methods:**

We retrieved positive blood cultures taken from patients in the Skåne region, southern Sweden, 2006–2019 from the Clinical Microbiology Department database and estimated incidence rates (IR), stratified by age (0–49, 50–64, 65–79, ≥ 80 years), sex, year, and species and described antimicrobial susceptibility for Enterobacterales.

**Results:**

We identified 944,375 blood culture sets, and 129,274 (13.7%) were positive. After deduplication and removal of contaminants, 54,498 separate BSI episodes remained. In total, 30,003 BSI episodes (55%) occurred in men. The overall IR of BSI was 307/100,000 person-years, with an average annual increase of 3.0%. Persons ≥ 80 years had the highest IR, 1781/100,000 person-years, as well as the largest increase. *Escherichia coli* (27%) and *Staphylococcus aureus* (13%) were the most frequent findings. The proportion of Enterobacterales isolates resistant to fluoroquinolones and third generation cephalosporins increased from 8.4% to 13.6%, and 4.9% to 7.3%, (p for trend < 0.001), with the largest increase in the oldest age group.

**Conclusion:**

We report among the highest BSI IRs to date worldwide, with a higher proportion among elderly persons and males, including resistant isolates. Given expected demographic changes, these results indicate a possible substantial future BSI burden, for which preventive measures are needed.

**Key public health message**

**What did you want to address in this study?**

Bloodstream infections are a public health concern, causing an estimated 150,000 European deaths annually. Previous incidence estimates are inconsistent, possibly due to variations in methodology and definitions. We aimed to provide an updated, population-based estimate of the incidence of bloodstream infections and relate this to the forthcoming demographic changes, with an ageing population.

**What have we learnt from this study?**

We found a high incidence rate of bloodstream infections at 307 per 100,000 person-years. From 2006 to 2019, the average annual increase in incidence rate was 3.0%. The oldest age group (≥ 80 years) had the highest incidence rate and a disproportionate increase, including of isolates with antimicrobial resistance.

**What are the implications of your findings for public health?**

This population-based study reports among the highest incidence rates of bloodstream infections to date, with the highest incidence rates described in elderly persons. Given the projected ageing of the population, the burden of BSI on healthcare systems is likely to increase. Any preventive measures should prioritise the oldest patients.

## Introduction

Bloodstream infections (BSI) are a public health concern, causing an estimated 150,000 deaths in Europe annually [1]. Despite improvements in treatment and critical care, BSI remain a medical emergency, with a 30-day case-fatality rate ranging between 10 and 20% [2-5]. Infections caused by resistant bacteria have further increased the overall BSI burden, morbidity, and mortality [6]. Population projections suggest a considerable increase in the population aged over 65 years globally, in whom BSI incidence and mortality are the highest, emphasising the need for preventive measures [7].

Studies from the northern hemisphere published in the last decade are suggesting a BSI incidence rate (IR) of 150–250 per 100,000 person-years, with the majority of cases being community-acquired [2,3,8,9]. However, results vary substantially between studies; a recent Canadian study described an IR of 150 per 100,000 person-years in 2017, with a concurrent study from Finland presenting an IR of 309 per 100,000 person-years in 2018 [2,5]. This discrepancy in IR may be explained by differences in capturing (blood culture rates, methodology etc.) and reporting (how to define duplicates and contaminations) of BSI incidence [10]. Firstly, blood culturing rates and culture methodology depend on local practice and resources. Secondly, different definitions of contaminations and relapse bacteraemia are used. Lack of age-standardisation complicates comparisons across populations with different age compositions. Moreover, estimates from hospital-based studies may differ from population-based estimates, due to geographical aspects and the organisation of healthcare [10].

The aim of this study is to report incidence, aetiology and resistance patterns of bloodstream infections and their progression in southern Sweden between 2006 and 2019. The overall purpose is to provide an updated, population-based estimate and relate this to the anticipated demographic changes.

## Methods

### Study design and setting

This retrospective study used data from a microbiology database to describe BSI incidence from 1 January 2006 to 31 December 2019 in Skåne, southern Sweden, a region with ca 1.4 million inhabitants, see Supplementary material S1 for a description of geography and healthcare in the Skåne region. There is only one database for microbiological diagnostics in the region, which is at the Department of Clinical Microbiology in Lund. During the study period, local routine stated that two sets of blood cultures should be drawn from two separate venepunctures upon suspecting BSI. In the Skåne region, blood cultures are exclusively taken at hospitals providing secondary and tertiary healthcare, at emergency departments, inpatient wards or (rarely) in hospital-based outpatient care. At emergency departments, a nurse may obtain initial blood samples, including blood cultures, if suspecting BSI during triage. Whether the cultures are to be sent for analysis or not is then decided by the treating physician. The BacT/ALERT blood culture system (bioMérieux, Inc., Marcy-l’Étoile, France) was used in the Skåne region until December 2014, when it was replaced by the BACTEC FX (BectonDickinson, Franklin Lakes, United States). Susceptibility testing was performed by disk diffusion according to the European Committee on Antimicrobial Susceptibility Testing (EUCAST) standards [11].

### Data collection

Positive blood cultures were identified in the database and the sample date, age, sex and microbiological findings were retrieved. For species with clinically used names that were changed during the study period (such as *Cutibacterium acnes* previously called *Propionibacterium acnes*) we have consistently aimed to use the valid names as of 2022 according to the International Code of Nomenclature of Prokaryotes [12]. Antimicrobial susceptibility was described for Enterobacterales only, for fluoroquinolones (ciprofloxacin), third generation cephalosporins (cefotaxime) and aminoglycosides (gentamicin). Due to a database update in 2010, susceptibility data were available from 2011 to 2019 only. In addition, zone diameters were incomplete in > 50% of records. Therefore, clinical classification into susceptible (S), increased exposure (I) and resistant (R) was used to describe susceptibility, using breakpoints as per the original microbiology reports [13]. For cases with zone data, susceptibility testing according to the 2022 EUCAST breakpoints was compared with the original SIR classification in a sensitivity analysis [11]. Negative blood cultures were retrieved on an aggregate level (only the total number of blood culture sets per year was available). Population data were retrieved from Statistics Sweden [14].

### Definitions

One pair of bottles (aerobic + anaerobic) with a result in the microbiology database formed a blood culture set. A positive blood culture was defined as a blood culture set with one or more positive findings. Potential contaminants were bacteria that are part of the normal skin microbiota (e.g. Coagulase-negative staphylococci, *Corynebacterium, Cutibacterium*), see Supplementary material S2, Classification of potential contaminants for details. These were considered contaminants if only one blood culture set was positive within 48 hours.

The deduplication period, the period during which only one BSI episode was registered, was set to 14 days. As the deduplication period varies between previous studies, sensitivity analyses were performed for 30, 90 and 365 days. A duplicate was defined as a culture for which there was another positive blood culture with the same finding, taken within the deduplication period. The positive blood cultures remaining after removal of contaminations and duplicates were considered relevant findings.

A polymicrobial finding was two or more different relevant findings from the same patient, obtained within the deduplication period. A BSI episode was defined as an episode with at least one relevant finding and where polymicrobial findings are deduplicated. Thus, if a blood culture set simultaneously grew *Escherichia coli* and *Klebsiella* spp., this would count as two relevant findings but only one BSI episode. An R classification in the original microbiology report defined antimicrobial resistance.

### Statistical analysis

Crude incidence rates (IR) were determined by dividing the number of BSI episodes with the population of Skåne. To increase comparability and to adjust for changes in population structure over time, age-standardised rates were estimated using the direct standardisation method and the 2013 European standard population [15]. Due to the focus on the ageing population, age-specific rates were described in 0–49, 50–64, 65–79 and ≥ 80 year strata. Crude rates were used for sex stratified IRs. Differences in IRs were estimated using incidence rate ratios (IRR). All IRs are presented as cases per 100,000 person-years, and IR and IRRs are presented with 95% confidence intervals.

To model change in overall IR over time, we fitted segmented regressions with the number of BSI episodes as the dependent variable, age and year as independent variables and the natural log of population as offset [16]. The results are expressed as annual percent change (APC) for each segment as well as the average annual percent change (AAPC) for the entire period [17]. For hypothesis testing of differences in the proportion of resistant isolates across sexes, the chi-square test was used. To test trends in the proportion of resistant isolates, Cochrane-Armitage trend tests were used, using Bonferroni correction for multiple testing. A p value of <0.05 was considered significant.

## Results

### Blood cultures and BSI episodes

In total, 944,375 blood culture sets were registered, yielding an overall culturing rate of 5,322 sets per 100,000 person-years. Of these, 129,274 (13.7%) were positive, with a positivity rate that remained stable over time, see Supplementary Table T1, Population, blood cultures and findings 2006 - 2019. Forty-five positive blood culture sets (0.03%) were excluded due to incomplete data, leaving 129,229 for the analysis. From the positive cultures, 23,113 (17.9%) contaminants and 43,334 (33.5%) duplicates were removed, leaving 62,782 relevant findings. Of these, 8,284 polymicrobial findings (13.2%) were deduplicated, leaving 54,498 separate BSI episodes, in 43,421 individuals. Of these, 36,161 (83%) had one, 5,163 (12%) had two, and 2,097 (5%) had three or more BSI episodes during the 14-year study period.

**Figure 1:**
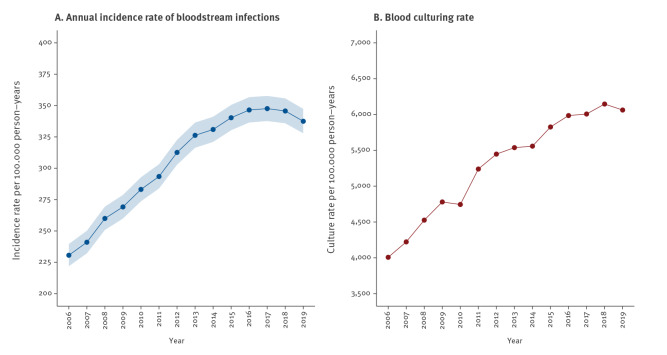
(A) Annual incidence rate of bloodstream infections and (B) blood culturing rate in southern Sweden, 2006–2019

Overall crude and age-standardised BSI incidence rates were both 307 (95% CI: 304 to 309) per 100,000 person-years, increasing from 231 in 2006 and peaking at 348 in 2017, see Figure 1. When de-duplication was extended to 30, 90 and 365 days in a sensitivity analysis, overall IR decreased to 298, 285 and 267, respectively. Segmented regression showed an increasing rate from 2006 to 2014, with an APC of 4.7% (95% CI: 4.3% to 5.2%). From 2014 to 2019 the slope was not significantly different from zero, APC: -0.2% (95% CI: -1.0% to 0.7%). For the entire period, the AAPC was 3.0% (95% CI: 2.8% to 3.2%).

Incidence rate has been standardised to the 2013 European standard population.

Shaded area represents 95% confidence interval.

### Age and sex

The median age was 73 years (IQR: 60–82) for the entire period, increasing from 72 years in 2006 to 74 in 2019. The IR was 1,781 per 100,000 person years (95% CI: 1,755 to 1,807) in those aged ≥ 80 years compared to an IR of 73 (95% CI: 72 to 75) in those aged 0–49 years, resulting in an IRR of 24.3 (95% CI: 23.7 to 24.9). In total, 30,003 BSI episodes (55%) occurred in men, and 24,495 (45%) in women. The IR for males was 341 per 100,000 person years (95% CI: 337 to 345) compared with 272 (95% CI: 268 to 275) for females, yielding an IRR of 1.25 (95% CI: 1.23 to 1.28).

**Figure 2:**
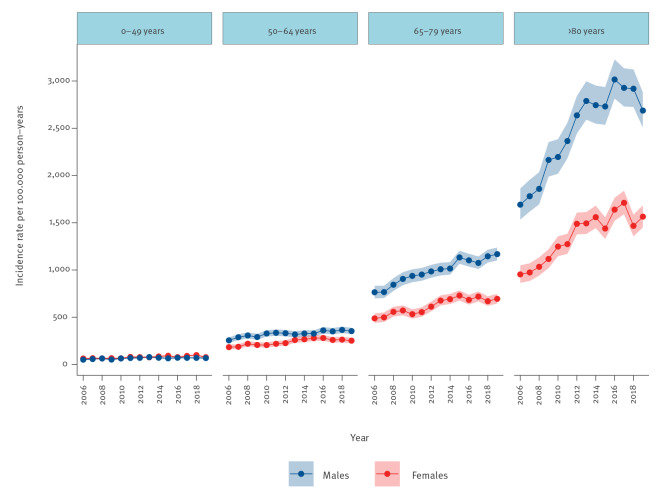
Incidence rate of bloodstream infections by age, sex, and year, southern Sweden, 2006–2019 (n = 54,498)

Over time, BSI incidence rates increased most in patients ≥ 80 years, from 1,219 per 100,000 person-years (95% CI: 1,138 to 1,304) in 2006 to 2,018 (95% CI: 1,917 to 2,122) in 2019. Consequentially, the difference between the oldest patients and the youngest, expressed as the IRR of BSI in ≥ 80 years vs 0–49 years, increased, from 19.9 (95% CI: 17.8 to 22.4) in 2006 to 28.0 (95% CI: 25.5 to 30.7) in 2019. Male BSI IR increased from 244 per 100,000 person years (95% CI: 232 to 257) in 2006 to 390 (95% CI: 376 to 405) in 2019. Female BSI incidence increased from 206 per 100,000 person years (95% CI: 195 to 217) in 2006 to 297 (95% CI: 284 to 310) in 2019. Thus, the difference between sexes, expressed as the IRR of males vs females, increased from 1.18 (95% CI: 1.10 to 1.28) to 1.32 (95% CI: 1.24 to 1.39). Incidence rates stratified by age and sex are shown in Figure 2.

Shaded area represents 95% confidence interval.

### Bacterial species

The most frequent bacteria found were *E. coli* and *Staphylococcus aureus*, isolated in 16,977 (27%) and 8,065 (13%) of relevant findings, respectively (Table). *E. coli* and *Klebsiella* spp*.* were more common in elderly people, with IRR for persons ≥ 80 years vs 0–49 years of 40.2 (95% CI: 38.2 to 42.4) and 39.1 (95% CI: 35.2 to 43.4) for the entire period. *Enterococcus* species had an IRR of 27.3 (95% CI: 24.5 to 30.5), all other species had an IRR of < 20. When stratified by sex, *Enterococcus* species stood out, being almost twice as frequent in males than females, with an IRR of 1.93 (95% CI: 1.81 to 2.07).

**Table:** Relevant bacterial findings by bacterial species, southern Sweden, 2006–2019 (n = 62,782)

Bacterial species	Number of relevant findings	% of all relevant findings	Age-standardised IR per 100,000 person-years	
			IR	95% CI
*Escherichia coli*	16,977	27.0	95.3	93.8–96.7
*Staphylococcus aureus*	8,065	12.8	45.4	44.4–46.4
*Streptococcus species*	7,712	12.3	43.3	42.3–44.2
*Staphylococcus species*	4,864	7.7	27.5	26.7–28.3
*Klebsiella species*	4,606	7.3	26.0	25.2–26.7
*Enterococcus species*	3,765	6.0	21.3	20.6–22.0
*Streptococcus pneumoniae*	2,792	4.4	15.8	15.2–16.4
Other	14,001	22.3	78.8	77.5–80.1

CI: confidence interval; IR: incident rate.

**Figure 3:**
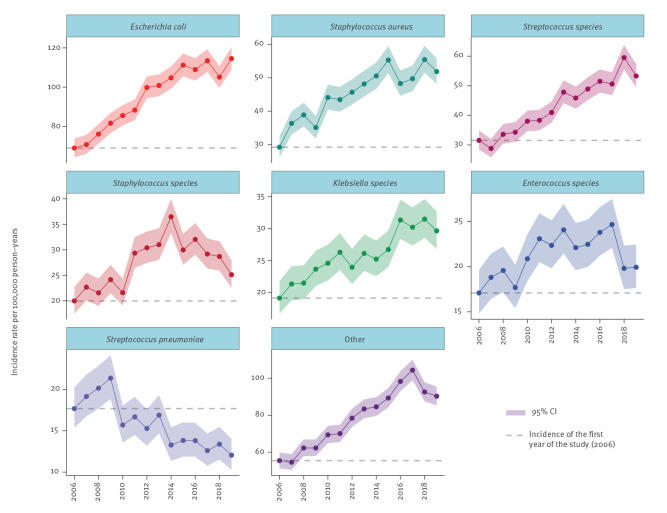
Incidence rate of positive blood cultures by bacterial species and year, southern Sweden, 2006–2019

Over time, the incidence rate increased for all species except *Streptococcus pneumoniae*, see Figure 3. The largest increase (measured as IRR of 2019 vs 2006) was found in *S. aureus*, *Streptococcus* species and *E. coli*. In persons ≥ 80 years, the IR increased the most for ’other’ bacteria, *Enterococcus* spp., and *S. aureus*, see Supplementary Figure F8. Incidence rate by species, age, sex and year.

*Streptococcus* species: all streptococci except *Streptococcus pneumoniae*; *Staphylococcus* species: all staphylococci except *Staphylococcus aureus*.

### Antimicrobial susceptibility

We identified 17,983 isolates from Enterobacterales, with 17,734 (98.6%) having resistance data in the original microbiology report. Of these, 2,617 (14.8%) were resistant to at least one of the three tested antibiotics with 1,951 (11.0%), 1,145 (6.5%), and 826 (4.7%) being resistant to ciprofloxacin, cefotaxime, and gentamicin, respectively. The sensitivity analysis showed a similar proportion of resistant isolates using the 2022 EUCAST breakpoints, see Supplementary Tables T2–T4. Upon age stratification, the largest proportion of resistant isolates was found in those aged 50–64 years, with 18.4% of isolates resistant to any antibiotic. The proportion of resistant isolates was larger in males than females, with 17.4% vs 11.9% for any antibiotic, 13.7% vs 8.1% for ciprofloxacin, 7.2% vs 5.7% for cefotaxime, and 5.3% vs 3.9% for gentamicin, respectively (chi-squared test with p < 0.001 for all comparisons).

**Figure 4:**
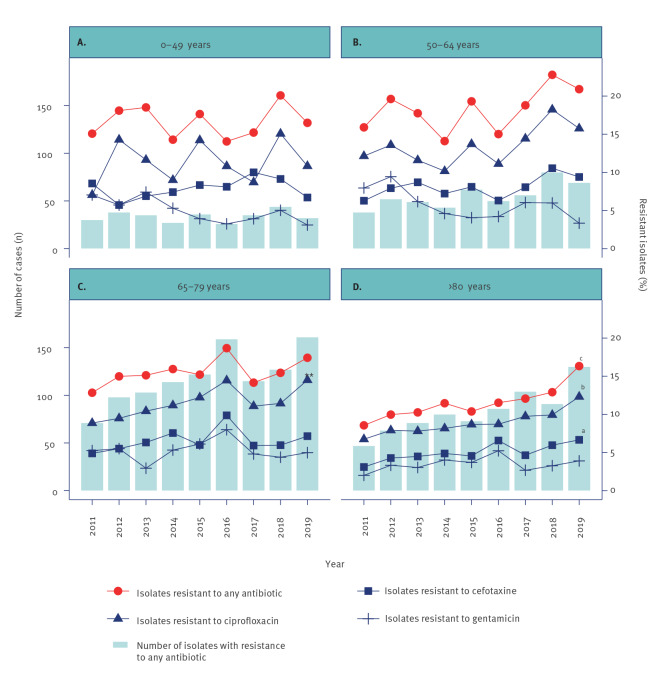
Enterobacterales isolates with antimicrobial resistance in (A) 0–49-year-olds, (B) 50–64-year-olds, (C) 65-79-year-olds and (D) 80-year-olds and older, southern Sweden, 2011–2019

^a^ p < 0.05.

^b^ p < 0.01.

^c^ p < 0.001.

Over time, the proportion of resistant isolates increased from 12.1% to 17.4% for any antibiotic, from 8.4% to 13.6% for ciprofloxacin and from 4.9% to 7.3% for cefotaxime (Cochran-Armitage test for trend, p < 0.001 in all) but did not change significantly for gentamicin. When stratified by age, the group aged ≥ 80 years had the largest increase in the proportion of resistant isolates, primarily for ciprofloxacin, see Figure 4. In males, the proportion of isolates resistant to any antibiotic increased significantly from 13.8% to 19.1% (Cohran-Armitage test for trend, p = 0.007), and from 10.3% to 16.0% for ciprofloxacin (Cohran-Armitage test for trend, p < 0.001). In females, there were significant increases in the proportion of isolates resistant to any antibiotic (from 10.2% to 15.5%), to ciprofloxacin (from 6.3% to 10.9%) and for cefotaxime (from 4.2 to 7.3%), (Cohran-Armitage test for trend, p < 0.001 for all).

All p values from Cochrane-Armitage tests, with Bonferroni correction for multiple comparisons.

## Discussion

This study reports an overall BSI incidence rate of 307 per 100,000 person-years in southern Sweden during the period 2006–2019, with incidence rates increasing from 2006 to 2014, and then stabilising. The highest increase was found among persons ≥ 80 years and males, including that of resistant isolates.

The overall IR is among the highest reported, but comparisons require caution. We used a shorter deduplication period (14 days) than most other studies, and sensitivity analyses using 365 days led to an IR decrease from 307 to 267 per 100,000 person years. Without age-standardised estimates, differences in age composition of the study population may also complicate comparisons [10]. The patients in the present study were older (median age 73 years) than in previous reports, possibly indicating an older population, which may have a substantial impact as IRs increased exponentially with age [2-4]. In addition, culturing rates were high at 5,300 blood culture sets per 100,000 person-years, which may have affected results [18,19]. The local routine that ED nurses may obtain blood cultures before consulting a physician could have contributed to the high culturing, and incidence, rates. Three concurrent studies have reported culturing rates ranging between 4,000 and 5,000, with BSI incidence lower than in our study at 150, 185 and 223 per 100,000 person years, respectively [5,8,18]. A Danish study reported higher culturing rates but still lower IR at 216 per 100,000 person-years, although this study excluded recurrent bacteremias [9]. A period effect also seems to exist where comparisons with older studies may be less appropriate as more recent studies report higher IRs, such as the study from Finland with an IR of 309 per 100,000 person years in 2018 [2].

Our results suggested an increasing IR over time, in line with previous studies indicating a long-term increase of BSI IR, from 76 to 125 per 100,000 person years in the 1980s and 1990s, compared with 159 to 309 from 2000 and onwards [2-4,9,19,20]. However, there was also a concurrent increase in culturing rates and this relationship may be bidirectional; either an increased surveillance may have led to improved BSI identification, or an increase in severe infectious diseases could have entailed an increased culturing rate. Previous studies with increasing culturing rates have found a decreasing positivity rate, suggesting liberalised culturing indications [8,9,21]. In contrast, the positivity rate remained stable over 14 years in our study. Determining the ideal culture rate is challenging, but interestingly, the flattening of the IR trend in 2014 coincided with culturing rates reaching a previously suggested optimal level between 5,500 and 6,500 [18]. Apart from increased detection rates, the long-term increase in BSI incidence has been ascribed to lifestyle factors, longer disease survival and an increased prevalence of comorbidities and invasive procedures [2,4,8]. A nationwide study in Sweden presented increasing hospitalisation rates with infectious disease diagnoses from 1998 to 2019, suggesting an increased incidence of underlying severe infectious diseases [22]. However, due to the absence of clinical data, we were unable to discern to what extent culturing frequency vs host-related factors have contributed to the increasing IRs in our study.

The species distribution was similar to previous reports, with *E. coli* being the most frequently isolated pathogen, followed by *S. aureus* [2-4,8,9,23]. The IR for *E.coli* was higher than previously reported, with an IR of 115 per 100,000 person years in 2019, compared with IRs ranging from 28 to 74 in a recent systematic literature review, and 87 in a multinational population-based study [24,25]. The incidence of the other major pathogens found were also among the highest reported by others, although not as prominent as for *E. coli* [26-28]. Of the most common bacteria, a higher proportion of Gram-negative bacteria and enterococci were found among patients ≥ 80 years old, indicating that these may become more prevalent due to demographic change. The main source of bacteraemia with these pathogens is the urinary tract, and several predisposing conditions are common among elderly persons, including prostate disorders and urinary catheterisation [24]. Over time, IRs increased for all the major species except for *S. pneumoniae,* for which the decline was concurrent to the introduction of the paediatric pneumococcal vaccination programme in Sweden in 2009 [29].

Multidrug-resistant bacteria are quite rare in Sweden, but our results show increasing resistance to fluoroquinolones and cephalosporins for Enterobacterales, in line with previous studies [2,30]. The highest proportion of resistant isolates was found in patients aged 50–64 years, indicating that colonisation may be higher in this group. This is the age group turning 80 years old or older in the coming decades, the age where BSI incidence rises steeply. In patients colonised with extended spectrum beta-lactamase (ESBL)-producing bacteria, urinary tract infections carry the largest risk of causing ESBL-bacteraemia, emphasising the need for preventive measures targeting urinary tract infections [31]. Of the three tested antimicrobials, the proportion of isolates resistant to ciprofloxacin increased the most. Concurrently, there was a substantial decrease in the rate of fluoroquinolone prescription in pharmacies of the Skåne region, according to the National Prescribed Drug Register [32]. This suggests that in-hospital fluoroquinolone use may promote resistance, in line with a study from the United Kingdom that also indicated that hospital-based interventions could reverse this trend [33]. Finally, since the increase in antimicrobial resistance was most prominent among the patients >80 years, the overall impact of multidrug resistant BSIs could be expected to increase with the future demographic change.

The most important limitation is the lack of clinical data, preventing further classification of BSI episodes. As stated above, without clinical data, there is the possibility of ascertainment bias, with a higher detection of mild cases. In addition, the lack of granular data for negative cultures precluded an analysis of culture rates, positivity rates and trends within age strata. As the number of blood culture sets taken per patient was unknown, BSI episodes may have erroneously been classified as contaminations if only one set was taken (as often in paediatric patients), possibly leading to a minor underestimation of the total BSI incidence. Furthermore, individual residency status was not recorded, but hospital care in Skåne is almost exclusively provided to residents, as shown in Supplementary material S1, Setting - geography and healthcare in the Skåne region. The blood culturing system changed during the study period, which may have influenced our results. In addition, the breakpoints at the time of culture had to be used due to missing zone diameter data. Yet, the sensitivity analysis showed no indication of changes in breakpoints imitating trends.

Our results highlight the need for harmonisation in reporting BSI incidence, regarding local routines, contaminants, deduplication, culturing rates as well as age-standardisation. A sixth of patients had multiple BSI episodes, suggesting a need for studies identifying risk factors, possibly permitting secondary prevention. Future studies should focus on identifying the main factors underlying the seemingly general increase in BSI incidence. From a public health perspective, Swedish and European population projections unanimously suggest a considerable increase in persons aged ≥ 80 years in the coming decades. This age group had the highest BSI incidence as well as the largest increase in BSI incidence, including resistant isolates, which should make it a priority for preventive measures. Our results suggest that such measures should include those targeting urinary tract infections e.g. by minimising the use of urinary catheters and optimising UTI treatment and antimicrobial stewardship in urology departments.

### Conclusion

The results of this population-based study are among the highest reported BSI incidence rates to date, with the highest incidence rates described in persons ≥ 80 years. Given the projected ageing of the population, the burden of BSI on healthcare systems is likely to increase. Any preventive measures should prioritise the oldest patients.

## Supplementary Data

662000 Supplementary Material
