# Supplementary material for: Incidence, aetiology and temporal trend of bloodstream infections in southern Sweden from 2006 to 2019: a population-based study
Source: Euro Surveill. 2023 Mar 9;28(10):2200519. doi: 10.2807/1560-7917.ES.2023.28.10.2200519 (PMC9999458; doi:10.2807/1560-7917.ES.2023.28.10.2200519)
Supplement: Supplementary Material [file 22-00519_LJUNGQUIST_Supplement.pdf]

Supplementary appendix

This supplementary material is hosted by *Eurosurveillance* as supporting information alongside the article "**Incidence, aetiology, and temporal trend of bloodstream infections in southern Sweden from 2006 to 2019: a population-based study"**, on behalf of the authors, who remain responsible for the accuracy and appropriateness of the content. The same standards for ethics, copyright, attributions and permissions as for the article apply. Supplements are not edited by *Eurosurveillance* and the journal is not responsible for the maintenance of any links or email addresses provided therein.

S1. Setting – geography and healthcare in the Skåne region

The Skåne region is a peninsula in the southernmost part of Sweden, surrounded on three sides by the Baltic Sea. To the north, Skåne is neighbouring the regions of Halland, Kronoberg and Blekinge. In Skåne, most residents live in the southern and western parts.

In Skåne, ten hospitals provide acute inpatient care, covering almost all aspects of modern medicine (some highly specialised conditions are not treated in Skåne, e.g., liver transplants, severe burns) The placement of the hospitals entails that almost all Skåne residents have a hospital in Skåne as their nearest hospital, see map below. Thus, the absolute majority of Skåne residents would seek acute healthcare within the region. To confirm this, we retrieved the data for Skåne residents seeking healthcare outside Skåne from the regional reimbursement database. In total, 1.4% of hospital nights provided to Skåne residents was provided outside the region (in Sweden and abroad). Thus, 98.6% of hospital nights for Skåne residents was provided by hospitals in Skåne. This proportion was stable over time (varying between 98.4% and 98.7% annually).

In addition, some patients may travel to Skåne and receive care. This may be tourists or patients eligible for highly specialised care (e.g., specialised pediatric care, specialised surgery). These patients frequently stay for their immediate post-care in Skåne, after which they are transferred to their local hospital. To obtain an estimate of this, we also retrieved the number of hospital nights for non-residents hospitalised in Skåne from the regional reimbursement database. The proportion of all hospital nights provided by Skåne hospitals to non-residents was 4.2% during the entire study period. This was also stable (varying between 3.7% and 4.8%).

Figure F1. Map of population density and hospitals in southern Sweden.

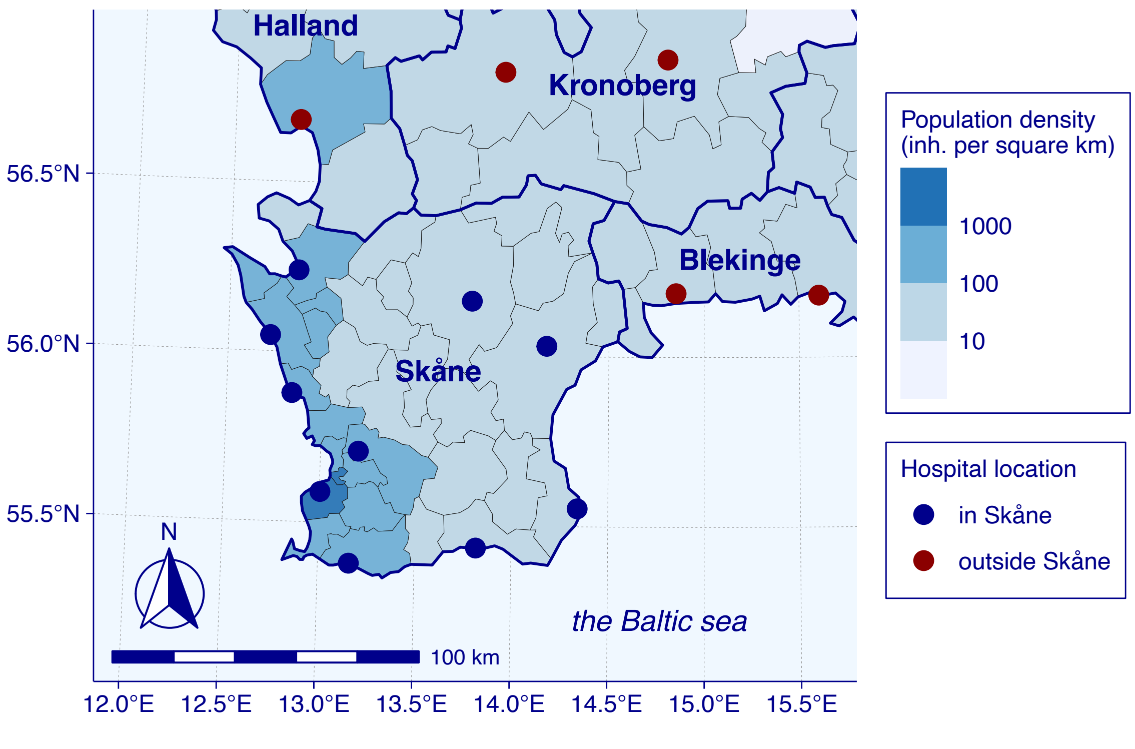

Figure F1. Population density in inhabitants per square kilometer. Skåne hospitals are marked in blue and those outside Skåne in red.

Comment

Even though we did not have information on individual residency status, we conclude that the geography and healthcare organisation of Skåne makes it suitable for population-based studies using hospital-based records for case-finding. The proportion of non-residents receiving care as well as the proportion of residents seeking healthcare outside Skåne was stable and unlikely to explain trends.

S2. Classification of potential contaminants

The choice was made to mainly restrict the classification of bacteria as potential contaminants to those known to be part of the skin flora, environmental bacteria, and those commonly regarded as being of limited pathogenicity. The following bacteria were considered as possible contaminants:

Bacillus, except for *Bacillus anthracis* (which was not found in the study).

Coagluase-negative *Staphylococcus* except for *S. lugdunensis*.

Cellulosimicrobium

Corynebacterium

Cutibacterium

Lactobacillus

Micrococcus, and other Micrococcales such as *Kocuria*, *Rothia*, *Dermabacter*, *Kytococcus*, etc.

Propionibacterium

Vagococcus

Findings that were only reported as skin flora, Gram negative environmental bacteria, Gram positive rods, Gram labile rods, as well as mixed bacterial flora were also considered possible contaminants.

The full classification is presented in supplementary table S5 at the end of this document.

Supplementary table T1. Population, blood cultures and findings 2006 - 2019

				positive		contamin.		duplicates		relevant		polymicrobial		BSI	
year	pyrs	cult.	pat.	n	%	n	%	n	%	n	%	n	%	n	rate
2 006	1 176 437	47 144	18 073	6 344	13	1 265	20	2 031	32	3 048	48	367	12	2 681	228
2 007	1 190 803	50 282	19 343	6 753	13	1 300	19	2 208	33	3 245	48	401	12	2 844	239
2 008	1 205 914	54 582	20 507	7 448	14	1 417	19	2 490	33	3 541	48	444	13	3 097	257
2 009	1 221 672	58 394	22 332	7 580	13	1 396	18	2 516	33	3 668	48	419	11	3 249	266
2 010	1 236 224	58 652	22 550	8 140	14	1 523	19	2 663	33	3 954	49	481	12	3 473	281
2 011	1 247 338	65 353	24 154	8 694	13	1 661	19	2 847	33	4 186	48	544	13	3 642	292
2 012	1 256 922	68 471	25 271	9 196	13	1 734	19	2 976	32	4 486	49	554	12	3 932	313
2 013	1 266 994	70 164	25 986	9 901	14	1 864	19	3 243	33	4 794	48	650	14	4 144	327
2 014	1 280 257	71 161	25 903	10 474	15	2 114	20	3 459	33	4 901	47	636	13	4 265	333
2 015	1 294 687	75 428	27 165	10 354	14	1 695	16	3 509	34	5 150	50	720	14	4 430	342
2 016	1 310 901	78 460	28 007	10 919	14	1 845	17	3 725	34	5 349	49	751	14	4 598	351
2 017	1 333 646	80 113	28 335	11 395	14	1 922	17	3 940	35	5 533	49	835	15	4 698	352
2 018	1 352 802	83 145	28 893	11 140	13	1 748	16	3 899	35	5 493	49	756	14	4 737	350
2 019	1 369 456	83 026	28 889	10 891	13	1 629	15	3 828	35	5 434	50	726	13	4 708	344
ALL	17 744 053	944 375	345 408	129 229	13.7	21 113	17.9	43 334	33.5	62 782	48.6	8 284	13.2	54 498	307

Table T1. pyrs. = person-years, cult. = number of blood culture sets taken, pat. = individual patients with a blood culture that year, positive = positive blood culture sets (% = percent of culture sets), contamin. = contaminations (% = percent of positive), duplicates = duplicate findings (% of positive), relevant = relevant findings (% of positive), polymicrobial = polymicrobial cultures (% of relevant findings), BSI = number of BSI episodes (rate = crude rate per 100.000 person-years = BSI episodes / person-years * 100.000).

S3. Antimicrobial susceptibility – Sensitivity analysis of clinical microbiology reports vs 2022 EUCAST breakpoints

Our initial intention was to assess susceptibility using zone diameters /gradients and the 2022 EUCAST guidelines. However, due to database inconsistencies, there was a large, and increasing, degree of missing data regarding zone diameters. On the other hand, data regarding the SIR classification from the original microbiology report was almost 100% complete. These are compared below:

Table T2. Quinolone (ciprofloxacin) resistance for *Enterobacterales*

		Isolates where SIR data according to original breakpoints were available				Isolates where data on zone diameters were also available					
								SIR according to EUCAST 2022 breakpoints		SIR according to breakpoints used at time of isolation	
year	isolates	n	%	R	%R	n	%	R	%R	R	%R
2 011	1 624	1 551	96	132	8.5	1 185	73	85	7.2	82	6.9
2 012	1 787	1 764	99	179	10.1	1 313	73	131	10.0	131	10.0
2 013	1 866	1 844	99	179	9.7	1 285	69	134	10.4	128	10.0
2 014	1 938	1 909	99	185	9.7	1 079	56	114	10.6	111	10.3
2 015	2 051	2 031	99	232	11.4	1 128	55	140	12.4	134	11.9
2 016	2 126	2 115	99	245	11.6	563	26	82	14.6	77	13.7
2 017	2 212	2 202	100	237	10.8	541	24	100	18.5	105	19.4
2 018	2 113	2 099	99	261	12.4	516	24	114	22.1	139	26.9
2 019	2 266	2 245	99	305	13.6	565	25	121	21.4	127	22.5

Table T2. Isolates = number of relevant *Enterobacterales* findings, n = number of isolates with available data (SIR from microbiology report (left) and zone diameters (right), %= percent of all isolates with available data, R = number of isolates classified as R, %R = percentage of classified isolates that were classified as R.

Table T3. Cephalosporin (cefotaxime) resistance for *Enterobacterales*

		Isolates where SIR data according to original breakpoints were available				Isolates where data on zone diameters were also available					
								SIR according to EUCAST 2022 breakpoints		SIR according to breakpoints used at time of isolation	
year	isolates	n	%	R	%R	n	%	R	%R	R	%R
2 011	1 624	1 608	99	81	5.0	1 308	81	59	4.5	63	4.8
2 012	1 787	1 771	99	97	5.5	1 316	74	67	5.1	71	5.4
2 013	1 866	1 844	99	111	6.0	1 288	69	87	6.8	90	7.0
2 014	1 938	1 915	99	124	6.5	1 080	56	82	7.6	82	7.6
2 015	2 051	2 035	99	123	6.0	1 122	55	72	6.4	72	6.4
2 016	2 126	2 115	99	169	8.0	555	26	71	12.8	70	12.6
2 017	2 212	2 202	100	135	6.1	520	24	96	18.5	96	18.5
2 018	2 113	2 099	99	148	7.1	509	24	134	26.3	134	26.3
2 019	2 266	2 244	99	163	7.3	561	25	142	25.3	142	25.3

Table T3. Isolates = number of relevant *Enterobacterales* findings, n = number of isolates with available data (SIR from microbiology report (left) and zone diameters (right), %= percent of all isolates with available data, R = number of isolates classified as R, %R = percentage of classified isolates that were classified as R.

Table T4. Aminoglycoside (gentamicin) resistance for *Enterobacterales*

		Isolates where SIR data according to original breakpoints were available				Isolates where data on zone diameters were also available					
								SIR according to EUCAST 2022 breakpoints		SIR according to breakpoints used at time of isolation	
year	isolates	n	%	R	%R	n	%	R	%R	R	%R
2 011	1 624	1 599	98	74	4.6	1 098	68	45	4.1	47	4.3
2 012	1 787	1 770	99	95	5.4	1 170	65	72	6.2	69	5.9
2 013	1 866	1 838	98	72	3.9	1 274	68	58	4.6	55	4.3
2 014	1 938	1 915	99	90	4.7	1 076	56	58	5.4	49	4.6
2 015	2 051	2 036	99	96	4.7	1 129	55	61	5.4	55	4.9
2 016	2 126	2 115	99	127	6.0	552	26	48	8.7	44	8.0
2 017	2 212	2 202	100	89	4.0	531	24	43	8.1	41	7.7
2 018	2 113	2 097	99	91	4.3	520	25	62	11.9	57	11.0
2 019	2 266	2 245	99	94	4.2	589	26	59	10.0	56	9.5

Table T4. Isolates = number of relevant *Enterobacterales* findings, n = number of isolates with available data (SIR from microbiology report (left) and zone diameters (right), %= percent of all isolates with available data, R = number of isolates classified as R, %R = percentage of classified isolates that were classified as R.

Comment

There was a substantial and increasing degree of missingness for zone data that was non-random. Antimicrobial resistance was much more common among isolates with zone data available. However, when the original microbiology reports were compared to zone data applying 2022 EUCAST breakpoints in isolates with both methods available, the results were quite similar, indicating an acceptable validity of the original reports.

S4. Supplementary figures

Figure F2. Flowchart of blood culture sets in Skåne region from 2006 to 2019

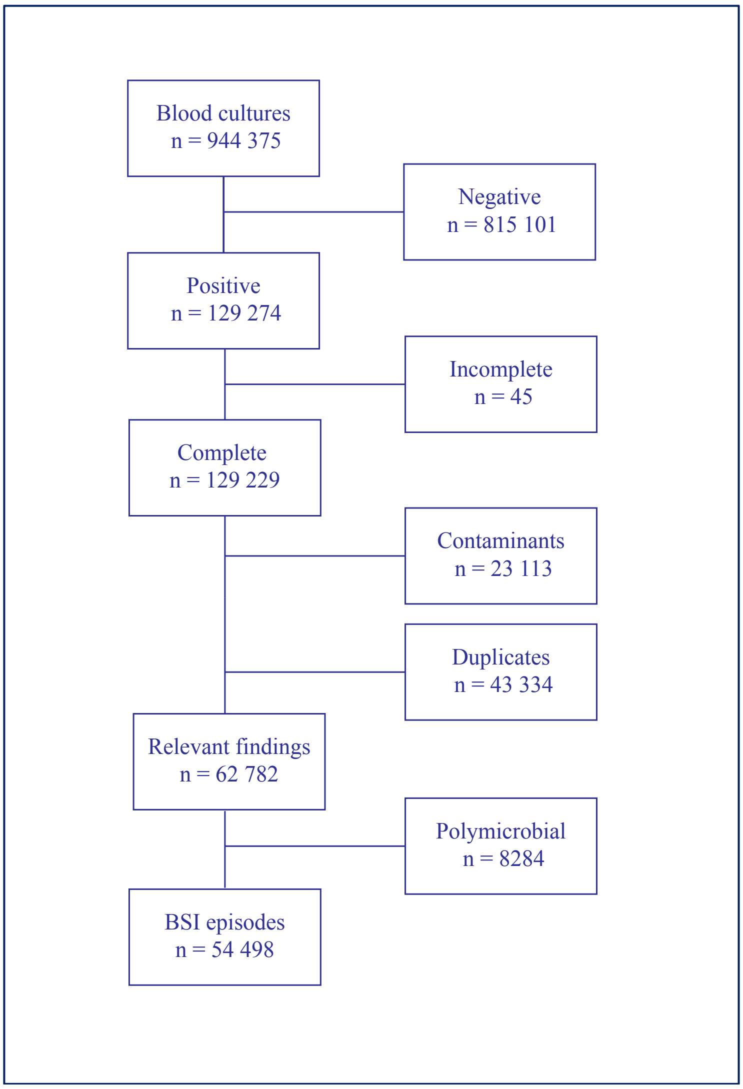

Figure F2. Flowchart from the database retrieval**.** All definitions are specified in the main manuscript. BSI = bloodstream infection.

Figure F3. Crude and age-standardized BSI incidence rates, by year.

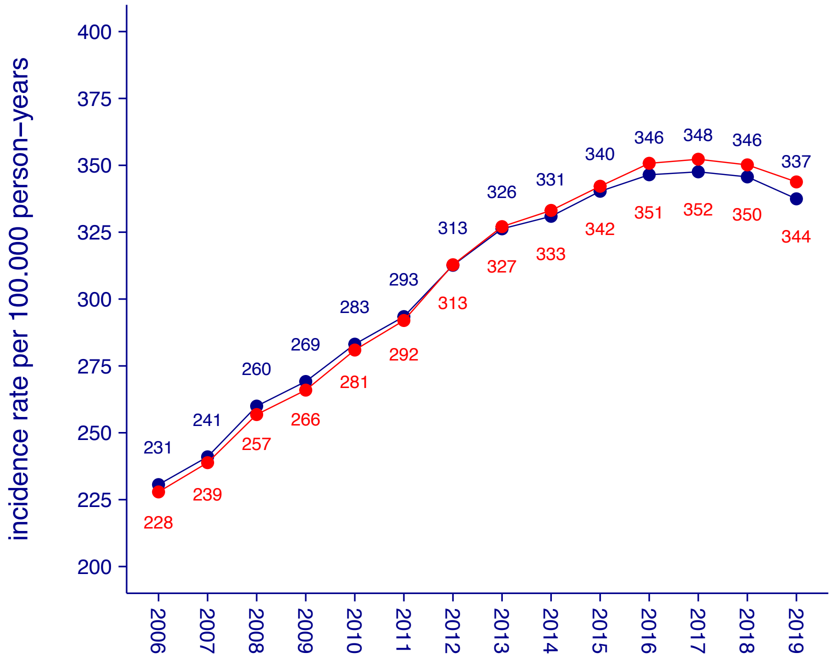

Figure F3. Blue dots, lines, numbers = point estimates of age-standardized rates (standardized to the 2013 European standard population, using the direct method). Red dots, lines, and numbers = point estimates of crude incidence rates.

Figure F4. BSI Incidence rate with segmented regression fit.

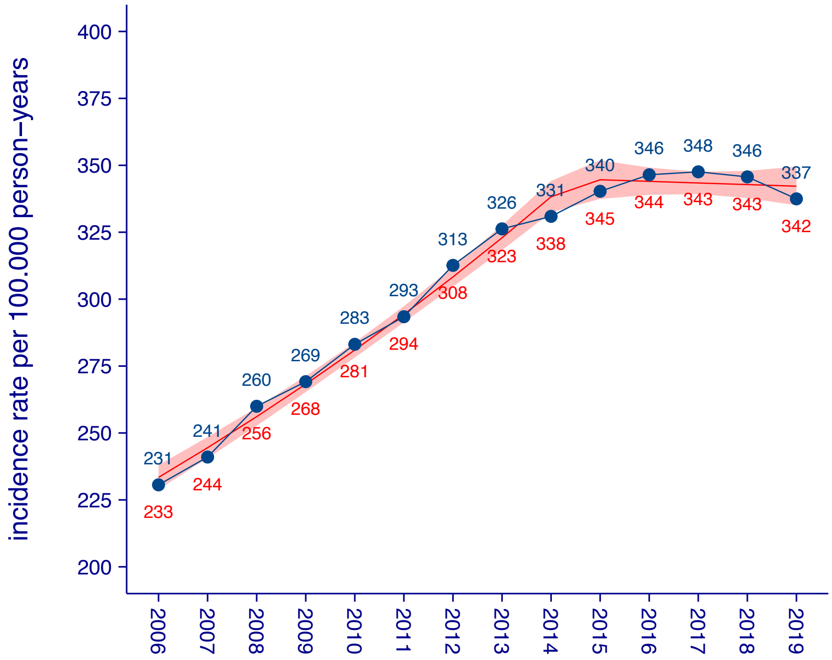

Figure F4. Blue dots, lines, numbers = point estimates for age-standardized incidence rate. Red line, numbers = point estimates from segmented regression fit. Shaded red area = 95% confidence interval from regression fit.

Figure F5. Number of BSI cases and incidence rate, by age and sex

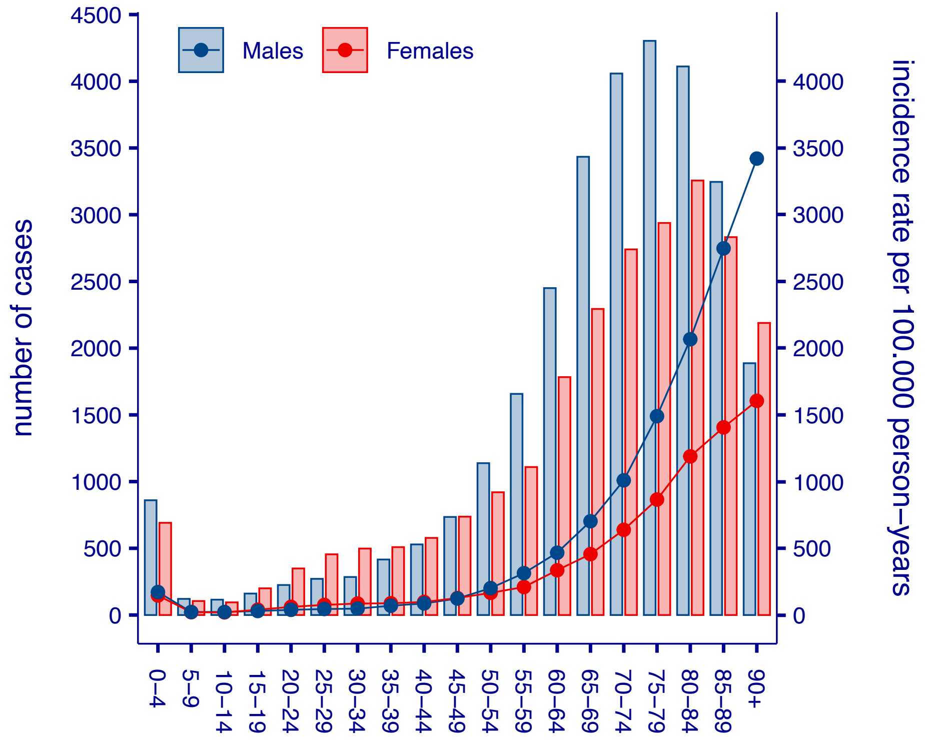

Figure F5. Bars represent cases (left y axis), and dots / lines represent incidence rate (right y axis).

Figure F6. BSI Incidence rate by sex and year

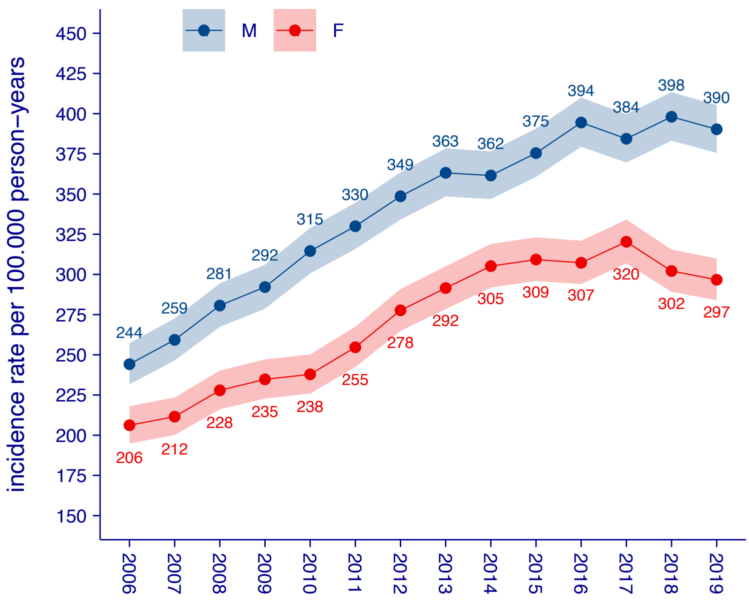

Figure F6. Rates are presented as crude incidence rates. Dots, lines, and numbers = point estimates, shaded area = 95% confidence interval.

Figure F7. Species distribution, by age and sex.

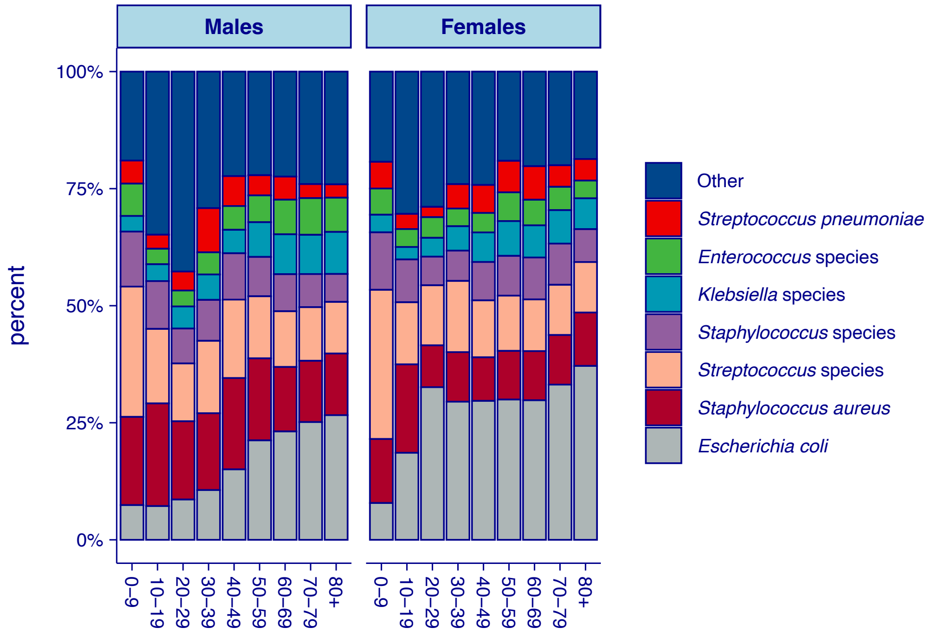

Figure F7. Species distribution for the seven most prevalent species and other by age strata and sex. *Streptococcus* species = all streptococci except *Streptococcus pneumoniae*, *Staphylococcus* species = all staphylococci except *Staphylococcus aureus*.

Figure F8. incidence rate by species, age, sex and year.

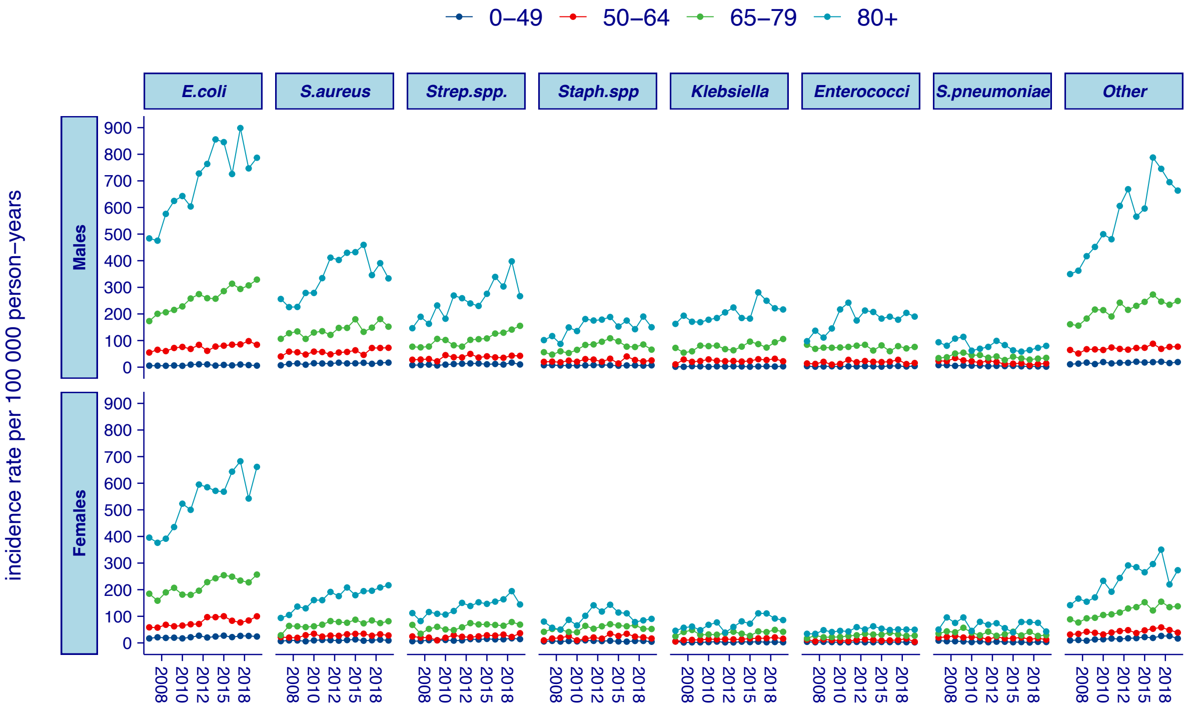

Figure F8. Age-specific incidence rates for the seven most prevalent species and a group of others. *Strep. spp.* = all streptococci except *Streptococcus pneumoniae*, *Staph. spp* = all staphylococci except *Staphylococcus aureus*.

Table T5. Species distribution of positive blood cultures (N = 129274)

Note: These are all positive findings, before any removal of contaminants or deduplication

Species	possible contaminant	count	percent	cumulative %
Escherichia coli	No	27892	21.6%	21.6%
Staphylococcus species	Yes	20388	15.8%	37.3%
Staphylococcus aureus	No	15376	11.9%	49.2%
Staphylococcus epidermidis	Yes	7145	5.5%	54.8%
Klebsiella pneumoniae	No	5074	3.9%	58.7%
Streptococcus pneumoniae	No	4768	3.7%	62.4%
Enterococcus faecalis	No	3728	2.9%	65.3%
Pseudomonas aeruginosa	No	2117	1.6%	66.9%
Streptococcus dysgalactiae	No	2033	1.6%	68.5%
Enterococcus faecium	No	1954	1.5%	70.0%
Klebsiella oxytoca	No	1932	1.5%	71.5%
Streptococcus mitis	No	1795	1.4%	72.9%
Proteus mirabilis	No	1779	1.4%	74.2%
Enterobacter cloacae	No	1773	1.4%	75.6%
Bacteroides fragilis	No	1722	1.3%	76.9%
Streptococcus pyogenes	No	1666	1.3%	78.2%
Staphylococcus hominis	Yes	1494	1.2%	79.4%
Streptococcus anginosus	No	1442	1.1%	80.5%
Streptococcus species	No	1425	1.1%	81.6%
Streptococcus agalactiae	No	1357	1.0%	82.7%
Micrococcus species	Yes	1009	0.8%	83.4%
Corynebacterium species	Yes	984	0.8%	84.2%
Candida albicans	No	969	0.7%	85.0%
Staphylococcus capitis	Yes	654	0.5%	85.5%
Streptococcus salivarius	No	592	0.5%	85.9%
Serratia marcescens	No	586	0.5%	86.4%
Haemophilus influenzae	No	547	0.4%	86.8%
Cutibacterium acnes	Yes	540	0.4%	87.2%
Clostridium perfringens	No	512	0.4%	87.6%
Klebsiella aerogenes	No	478	0.4%	88.0%
Citrobacter freundii	No	441	0.3%	88.3%
Streptococcus bovis	No	437	0.3%	88.7%
Candida glabrata	No	365	0.3%	88.9%
Aerococcus urinae	No	363	0.3%	89.2%
Yeast	No	353	0.3%	89.5%
Morganella morganii	No	314	0.2%	89.7%
Bacillus cereus	Yes	313	0.2%	90.0%
Staphylococcus lugdunensis	No	307	0.2%	90.2%
Stenotrophomonas maltophilia	No	303	0.2%	90.4%
Bacillus species	Yes	288	0.2%	90.7%
Lactobacillus species	Yes	275	0.2%	90.9%
Citrobacter koseri	No	259	0.2%	91.1%
Streptococcus sanguinis	No	254	0.2%	91.3%
Acinetobacter species	No	240	0.2%	91.5%
Listeria monocytogenes	No	239	0.2%	91.7%
Bacteroides thetaiotaomicron	No	231	0.2%	91.8%
Parvimonas micra	No	224	0.2%	92.0%
Streptococcus mutans	No	220	0.2%	92.2%
Staphylococcus haemolyticus	Yes	219	0.2%	92.3%
Anaerobic gramnegative rods	No	186	0.1%	92.5%
Clostridium septicum	No	182	0.1%	92.6%
Proteus vulgaris	No	180	0.1%	92.8%
Eggerthella lenta	No	177	0.1%	92.9%

Neisseria meningitidis	No	150	0.1%	93.0%
Clostridium species	No	146	0.1%	93.1%
Cutibacterium species	Yes	138	0.1%	93.2%
Anaerobic grampositive cocci	No	132	0.1%	93.3%
Candida parapsilosis	No	131	0.1%	93.4%
Granulicatella adiacens	No	130	0.1%	93.5%
Anaerococcus species	No	129	0.1%	93.6%
Actinotignum schaalii	No	127	0.1%	93.7%
Fusobacterium necrophorum	No	126	0.1%	93.8%
Micrococcus luteus	Yes	121	0.1%	93.9%
Difteroid rods	Yes	116	0.1%	94.0%
Salmonella enteritidis	No	116	0.1%	94.1%
Grampositive rods	Yes	114	0.1%	94.2%
Clostridium ramosum	No	113	0.1%	94.3%
Anaerobic grampositive rods	Yes	109	0.1%	94.4%
Actinomyces species	No	108	0.1%	94.5%
Aeromonas species	No	108	0.1%	94.5%
Peptoniphilus harei	No	108	0.1%	94.6%
Staphylococcus warneri	Yes	108	0.1%	94.7%
Streptococcus parasanguinis	No	103	0.1%	94.8%
Fusobacterium nucleatum	No	102	0.1%	94.9%
Paenibacillus species	No	98	0.1%	94.9%
Rothia mucilaginosa	Yes	95	0.1%	95.0%
Citrobacter species	No	94	0.1%	95.1%
Bacteroides species	No	93	0.1%	95.2%
Pseudomonas species	No	93	0.1%	95.2%
Pasteurella multocida	No	90	0.1%	95.3%
Staphylococcus pettenkoferi	Yes	88	0.1%	95.4%
Veillonella species	No	88	0.1%	95.4%
Fusobacterium species	No	84	0.1%	95.5%
Salmonella typhi	No	84	0.1%	95.6%
Streptococcus gordonii	No	82	0.1%	95.6%
Neisseria species	No	79	0.1%	95.7%
Enterococcus casseliflavus	No	78	0.1%	95.8%
Capnocytophaga canimorsus	No	76	0.1%	95.8%
Gramnegative environmental bacte	Yes	71	0.1%	95.9%
Bacteroides vulgatus	No	69	0.1%	95.9%
Staphylococcus saprophyticus	Yes	69	0.1%	96.0%
Enterococcus gallinarum	No	68	0.1%	96.0%
Propionibacterium acnes	Yes	65	0.1%	96.1%
Grampositive cocci	No	63	0.0%	96.1%
Candida tropicalis	No	61	0.0%	96.2%
Enterococcus species	No	61	0.0%	96.2%
Anaerobic mixed growth	No	60	0.0%	96.3%
Providencia rettgeri	No	59	0.0%	96.3%
Parabacteroides distasonis	No	58	0.0%	96.4%
Aerococcus sanguinicola	No	57	0.0%	96.4%
Gemella morbillorum	No	57	0.0%	96.4%
Streptococcus constellatus	No	55	0.0%	96.5%
Bacteroides uniformis	No	54	0.0%	96.5%
Enterococcus avium	No	54	0.0%	96.6%
Peptostreptococcus species	No	53	0.0%	96.6%
Gemella species	No	51	0.0%	96.6%
Gramnegative rods	No	51	0.0%	96.7%
Corynebacterium striatum	Yes	49	0.0%	96.7%
Actinotignum species	No	48	0.0%	96.8%

Finegoldia magna	No	48	0.0%	96.8%
Hafnia alvei	No	47	0.0%	96.8%
Citrobacter diversus	No	46	0.0%	96.9%
Moraxella species	No	46	0.0%	96.9%
Streptococcus gallolyticus	No	46	0.0%	96.9%
Staphylococcus simulans	Yes	45	0.0%	97.0%
Streptococcus intermedius	No	45	0.0%	97.0%
Bacteroides ovatus	No	44	0.0%	97.0%
Haemophilus parainfluenzae	No	44	0.0%	97.1%
Bifidobacterium species	No	43	0.0%	97.1%
Candida krusei	No	43	0.0%	97.1%
Streptococcus vestibularis	No	43	0.0%	97.2%
Dermabacter hominis	Yes	42	0.0%	97.2%
Lactobacillus rhamnosus	Yes	42	0.0%	97.2%
Candida dubliniensis	No	41	0.0%	97.3%
Moraxella catarrhalis	No	41	0.0%	97.3%
Campylobacter jejuni	No	40	0.0%	97.3%
Prevotella species	No	40	0.0%	97.4%
Brevibacterium species	Yes	39	0.0%	97.4%
Acinetobacter baumannii	No	38	0.0%	97.4%
Enterobacter species	No	38	0.0%	97.5%
Staphylococcus caprae	Yes	37	0.0%	97.5%
Clostridium innocuum	No	36	0.0%	97.5%
Veillonella parvula	No	36	0.0%	97.5%
Moraxella osloensis	No	33	0.0%	97.6%
Klebsiella species	No	32	0.0%	97.6%
Rothia species	Yes	32	0.0%	97.6%
Actinomyces oris	No	30	0.0%	97.6%
Bacteroides ureolyticus	No	30	0.0%	97.7%
Kocuria species	Yes	30	0.0%	97.7%
Chryseobacterium indologenes	No	29	0.0%	97.7%
Microbacterium species	Yes	29	0.0%	97.7%
Candida pelliculosa	No	28	0.0%	97.8%
Dialister pneumosintes	No	28	0.0%	97.8%
Eikenella corrodens	No	28	0.0%	97.8%
Ruminococcus gnavus	No	28	0.0%	97.8%
Abiotrophia defectiva	No	27	0.0%	97.8%
Aerococcus viridans	No	27	0.0%	97.9%
Pseudomonas stutzeri	No	27	0.0%	97.9%
Salmonella grupp o 4	No	27	0.0%	97.9%
Achromobacter xylosoxidans	No	25	0.0%	97.9%
Rothia dentocariosa	Yes	25	0.0%	97.9%
Actinomyces odontolyticus	No	24	0.0%	98.0%
Peptoniphilus species	No	24	0.0%	98.0%
Candida lusitaniae	No	23	0.0%	98.0%
Peptostreptococcus micros	No	23	0.0%	98.0%
Gemella haemolysans	No	22	0.0%	98.0%
Salmonella paratyphi a	No	22	0.0%	98.1%
Sphingomonas paucimobilis	No	22	0.0%	98.1%
Proprionebacterium species	Yes	21	0.0%	98.1%
Actinomyces neuii	No	20	0.0%	98.1%
Atopobium species	No	20	0.0%	98.1%
Clostridium tertium	No	20	0.0%	98.1%
Dialister species	No	20	0.0%	98.1%
Brucella melitensis	No	19	0.0%	98.2%
Enterocloster clostridioformis	No	19	0.0%	98.2%

Enterococcus hirae	No	19	0.0%	98.2%
Pantoea agglomerans	No	19	0.0%	98.2%
Salmonella typhimurium	No	19	0.0%	98.2%
Bacteroides caccae	No	18	0.0%	98.2%
Gramnegative rods (enterobacteral	No	18	0.0%	98.2%
Lactobacillus gasseri	Yes	18	0.0%	98.3%
Lactococcus lactis	No	18	0.0%	98.3%
Peptostreptococcus asaccharolyticu	No	18	0.0%	98.3%
Salmonella grupp o 7	No	18	0.0%	98.3%
Salmonella paratyphi b	No	18	0.0%	98.3%
Vibrio vulnificus	No	18	0.0%	98.3%
Actinomyces turicensis	No	17	0.0%	98.3%
Aerococcus species	No	17	0.0%	98.4%
Campylobacter ureolyticus	No	17	0.0%	98.4%
Capnocytophaga sputigena	No	17	0.0%	98.4%
Eggerthella species	No	17	0.0%	98.4%
Odoribacter splanchnicus	No	17	0.0%	98.4%
Serratia species	No	17	0.0%	98.4%
Streptococcus equi	No	17	0.0%	98.4%
Aeromonas hydrophila	No	16	0.0%	98.4%
Anaerobic bacteria	No	16	0.0%	98.5%
Clostridium symbiosum	No	16	0.0%	98.5%
Eubacterium species	No	16	0.0%	98.5%
Flavonifractor plautii	No	16	0.0%	98.5%
Lactococcus garvieae	No	16	0.0%	98.5%
Salmonella grupp o 9	No	16	0.0%	98.5%
Staphylococcus pasteuri	Yes	16	0.0%	98.5%
Staphylococcus saccharolyticus	Yes	16	0.0%	98.5%
Streptococcus canis	No	16	0.0%	98.6%
Achromobacter species	No	15	0.0%	98.6%
Desulfovibrio species	No	15	0.0%	98.6%
Prevotella buccae	No	15	0.0%	98.6%
Salmonella species	No	15	0.0%	98.6%
Aggregatibacter aphrophilus	No	14	0.0%	98.6%
Clostridium paraputrificum	No	14	0.0%	98.6%
Moraxella nonliquefaciens	No	14	0.0%	98.6%
Pantoea species	No	14	0.0%	98.6%
Rhodococcus equi	No	14	0.0%	98.7%
Salmonella virchow	No	14	0.0%	98.7%
Yersinia enterocolitica	No	14	0.0%	98.7%
Acinetobacter ursingii	No	13	0.0%	98.7%
Brevundimonas species	No	13	0.0%	98.7%
Capnocytophaga species	No	13	0.0%	98.7%
Chryseobacterium species	No	13	0.0%	98.7%
Corynebacterium jeikeium	Yes	13	0.0%	98.7%
Desulfovibrio desulfuricans	No	13	0.0%	98.7%
Enterococcus durans	No	13	0.0%	98.8%
Gardnerella vaginalis	No	13	0.0%	98.8%
Globicatella species	No	13	0.0%	98.8%
Mixed growth of enterobacterales	No	13	0.0%	98.8%
Prevotella bivia	No	13	0.0%	98.8%
Salmonella java	No	13	0.0%	98.8%
Streptococcus sanguis	No	13	0.0%	98.8%
Actinobaculum schaalii	No	12	0.0%	98.8%
Alistipes onderdonki	No	12	0.0%	98.8%
Anaerobic gramnegative cocci	No	12	0.0%	98.8%

Clostridium cadaveris	No	12	0.0%	98.8%
Corynebacterium amycolatum	Yes	12	0.0%	98.9%
Hungatella hathewayi	No	12	0.0%	98.9%
Providencia stuartii	No	12	0.0%	98.9%
Staphylococcus sciuri	Yes	12	0.0%	98.9%
Streptococcus massiliensis	No	12	0.0%	98.9%
Streptococcus pasteurianus	No	12	0.0%	98.9%
Streptococcus thermophilus	No	12	0.0%	98.9%
Turicibacter sanguinis	No	12	0.0%	98.9%
Aeromonas caviae	No	11	0.0%	98.9%
Aggregatibacter actinomycetemcon	No	11	0.0%	98.9%
Arthrobacter species	No	11	0.0%	98.9%
Campylobacter species	No	11	0.0%	99.0%
Cardiobacterium species	No	11	0.0%	99.0%
Catabacter hongkongensis	No	11	0.0%	99.0%
Corynebacterium macginleyi	Yes	11	0.0%	99.0%
Gramvolatile rods	Yes	11	0.0%	99.0%
Kingella kingae	No	11	0.0%	99.0%
Lactobacillus plantarum	Yes	11	0.0%	99.0%
Lactococcus species	No	11	0.0%	99.0%
Salmonella dublin	No	11	0.0%	99.0%
Skin flora	Yes	11	0.0%	99.0%
Staphylococcus schleiferi	Yes	11	0.0%	99.0%
Streptococcus lutetiensis	No	11	0.0%	99.1%
Delftia acidovorans	No	10	0.0%	99.1%
Enterococcus raffinosus	No	10	0.0%	99.1%
Parabacteroides species	No	10	0.0%	99.1%
Salmonella stanley	No	10	0.0%	99.1%
Serratia liquefaciens	No	10	0.0%	99.1%
Solobacterium moorei	No	10	0.0%	99.1%
Sphingomonas species	No	10	0.0%	99.1%
Fusarium species	No	9	0.0%	99.1%
Nocardia farcinica	No	9	0.0%	99.1%
Peptoniphilus asaccharolyticus	No	9	0.0%	99.1%
Porphyromonas species	No	9	0.0%	99.1%
Proteus species	No	9	0.0%	99.1%
Pseudomonas putida	No	9	0.0%	99.1%
Raoultella ornithinolytica	No	9	0.0%	99.2%
Streptococcus oralis	No	9	0.0%	99.2%
Actinotignum urinale	No	8	0.0%	99.2%
Alcaligenes faecalis	No	8	0.0%	99.2%
Bifidobacterium breve	No	8	0.0%	99.2%
Brucella species	No	8	0.0%	99.2%
Candida species	No	8	0.0%	99.2%
Citrobacter braakii	No	8	0.0%	99.2%
Comamonas species	No	8	0.0%	99.2%
Cronobacter sakazakii	No	8	0.0%	99.2%
Leuconostoc citreum	No	8	0.0%	99.2%
Leuconostoc species	No	8	0.0%	99.2%
Neisseria flavescens	No	8	0.0%	99.2%
Ochrobactrum species	No	8	0.0%	99.2%
Propionimicrobium lymphophilum	Yes	8	0.0%	99.2%
Rhizobium radiobacter	No	8	0.0%	99.2%
Salmonella grupp o 8	No	8	0.0%	99.3%
Staphylococcus cohnii	Yes	8	0.0%	99.3%
Acinetobacter lwoffii	No	7	0.0%	99.3%

Atopobium rimae	No	7	0.0%	99.3%
Bilophila wadsworthia	No	7	0.0%	99.3%
Cardiobacterium hominis	No	7	0.0%	99.3%
Collinsella aerofaciens	No	7	0.0%	99.3%
Corynebacterium pseudodiphtheriti	Yes	7	0.0%	99.3%
Dolosigranulum pigrum	No	7	0.0%	99.3%
Facklamia hominis	No	7	0.0%	99.3%
Gemella sanguinis	No	7	0.0%	99.3%
Granulicatella species	No	7	0.0%	99.3%
Klebsiella variicola	No	7	0.0%	99.3%
Leclercia adecarboxylata	No	7	0.0%	99.3%
Leuconostoc lactis	No	7	0.0%	99.3%
Microaerophilic streptococci	No	7	0.0%	99.3%
Ochrobactrum anthropi	No	7	0.0%	99.3%
Paeniclostridium sordellii	No	7	0.0%	99.3%
Porphyromonas asaccharolytica	No	7	0.0%	99.4%
Psychrobacter species	No	7	0.0%	99.4%
Raoultella planticola	No	7	0.0%	99.4%
Salmonella subspecies i	No	7	0.0%	99.4%
Actinomyces naeslundii	No	6	0.0%	99.4%
Actinotignum sanguinis	No	6	0.0%	99.4%
Bacteroides intestinalis	No	6	0.0%	99.4%
Bacteroides stercoris	No	6	0.0%	99.4%
Brevibacillus species	No	6	0.0%	99.4%
Clostridioides difficile	No	6	0.0%	99.4%
Clostridium subterminale	No	6	0.0%	99.4%
Cryptococcus neoformans	No	6	0.0%	99.4%
Dialister micraerophilus	No	6	0.0%	99.4%
Gemella bergeri	No	6	0.0%	99.4%
Leptotrichia trevisanii	No	6	0.0%	99.4%
Prevotella denticola	No	6	0.0%	99.4%
Prevotella nigrescens	No	6	0.0%	99.4%
Providencia alcalifaciens	No	6	0.0%	99.4%
Providencia species	No	6	0.0%	99.4%
Saccharomyces cerevisiae	No	6	0.0%	99.4%
Salmonella (grupp b)	No	6	0.0%	99.4%
Sphingobacterium species	No	6	0.0%	99.5%
Acinetobacter johnsonii	No	5	0.0%	99.5%
Alistipes finegoldii	No	5	0.0%	99.5%
Anaerobic gramnegative mixed gro	No	5	0.0%	99.5%
Anaerobic grampositive mixed grov	No	5	0.0%	99.5%
Bacillus licheniformis	Yes	5	0.0%	99.5%
Clostridium aldenense	No	5	0.0%	99.5%
Clostridium clostridioforme	No	5	0.0%	99.5%
Clostridium sordellii	No	5	0.0%	99.5%
Fusarium solani	No	5	0.0%	99.5%
Granulicatella elegans	No	5	0.0%	99.5%
Candida kefyr	No	5	0.0%	99.5%
Kytococcus schroeteri	Yes	5	0.0%	99.5%
Leuconostoc mesenteroides	No	5	0.0%	99.5%
Microbacterium lacticum	No	5	0.0%	99.5%
Pediococcus pentosaceus	No	5	0.0%	99.5%
Peptostreptococcus stomatis	No	5	0.0%	99.5%
Salmonella (grupp c)	No	5	0.0%	99.5%
Salmonella heidelberg	No	5	0.0%	99.5%
Salmonella newport	No	5	0.0%	99.5%

Salmonella panama	No	5	0.0%	99.5%
Slackia exigua	No	5	0.0%	99.5%
Streptococcus cristatus	No	5	0.0%	99.5%
Streptococcus parasanguis	No	5	0.0%	99.5%
Sutterella wadsworthensis	No	5	0.0%	99.5%
Varibaculum species	No	5	0.0%	99.5%
Actinobaculum species	No	4	0.0%	99.6%
Actinomyces meyeri	No	4	0.0%	99.6%
Aggregatibacter segnis	No	4	0.0%	99.6%
Aggregatibacter species	No	4	0.0%	99.6%
Bacillus pumilus	Yes	4	0.0%	99.6%
Bacteroides pyogenes	No	4	0.0%	99.6%
Bifidobacterium longum	No	4	0.0%	99.6%
Brachybacterium species	No	4	0.0%	99.6%
Bulleidia extructa	No	4	0.0%	99.6%
Citrobacter amalonaticus	No	4	0.0%	99.6%
Comamonas kerstersii	No	4	0.0%	99.6%
Escherichia hermannii	No	4	0.0%	99.6%
Facklamia species	No	4	0.0%	99.6%
Fusobacterium gonidiaformans	No	4	0.0%	99.6%
Gordonia species	No	4	0.0%	99.6%
Gramnegative cocci	No	4	0.0%	99.6%
Kluyvera ascorbata	No	4	0.0%	99.6%
Leptotrichia species	No	4	0.0%	99.6%
Listeria species	No	4	0.0%	99.6%
Malassezia pachydermatis	No	4	0.0%	99.6%
Moraxella atlantae	No	4	0.0%	99.6%
Mycobacterium fortuitum	No	4	0.0%	99.6%
Mycobacterium goodii	No	4	0.0%	99.6%
Neisseria cinerea	No	4	0.0%	99.6%
Oral flora	Yes	4	0.0%	99.6%
Pediococcus species	No	4	0.0%	99.6%
Peptococcus species	No	4	0.0%	99.6%
Pluralibacter gergoviae	No	4	0.0%	99.6%
Prevotella melaninogenica	No	4	0.0%	99.6%
Rhodococcus species	No	4	0.0%	99.6%
Salmonella (grupp d)	No	4	0.0%	99.6%
Salmonella muenchen	No	4	0.0%	99.6%
Salmonella poona	No	4	0.0%	99.7%
Salmonella saintpaul	No	4	0.0%	99.7%
Salmonella thompson	No	4	0.0%	99.7%
Shewanella art	No	4	0.0%	99.7%
Shewanella species	No	4	0.0%	99.7%
Sneathia amnii	No	4	0.0%	99.7%
Streptobacillus moniliformis	No	4	0.0%	99.7%
Streptococcus suis	No	4	0.0%	99.7%
Tissierella praeacuta	No	4	0.0%	99.7%
Trueperella bernardiae	No	4	0.0%	99.7%
Vagococcus fluvialis	Yes	4	0.0%	99.7%
Actinomyces radicidentis	No	3	0.0%	99.7%
Actinomyces radingae	No	3	0.0%	99.7%
Anaerotruncus colihominis	No	3	0.0%	99.7%
Arcanobacterium haemolyticum	No	3	0.0%	99.7%
Bacillus simplex	Yes	3	0.0%	99.7%
Bacillus subtilis	Yes	3	0.0%	99.7%
Bordetella species	No	3	0.0%	99.7%

Burkholderia cepacia	No	3	0.0%	99.7%
Burkholderia multivorans	No	3	0.0%	99.7%
Butyricimonas species	No	3	0.0%	99.7%
Clostridium sporogenes	No	3	0.0%	99.7%
Corynebacterium minutissimum	Yes	3	0.0%	99.7%
Cupriavidus pauculus	No	3	0.0%	99.7%
Cutibacterium granulosum	Yes	3	0.0%	99.7%
Deinococcus species	No	3	0.0%	99.7%
Erysipelothrix rhusiopathiae	No	3	0.0%	99.7%
Escherichia vulneris	No	3	0.0%	99.7%
Eubacterium limosum	No	3	0.0%	99.7%
Exiguobacterium species	No	3	0.0%	99.7%
Fusarium verticillioides	No	3	0.0%	99.7%
Gramnegative mixed flora	No	3	0.0%	99.7%
Haemophilus aphrophilus	No	3	0.0%	99.7%
Helicobacter canis	No	3	0.0%	99.7%
Lelliottia amnigena	No	3	0.0%	99.7%
Lysinibacillus fusiformis	No	3	0.0%	99.7%
Lysinibacillus species	No	3	0.0%	99.7%
Neisseria elongata	No	3	0.0%	99.7%
Nocardia species	No	3	0.0%	99.7%
Paracoccus yeei	No	3	0.0%	99.7%
Parvimonas species	No	3	0.0%	99.8%
Pasteurella canis	No	3	0.0%	99.8%
Peptostreptococcus prevotii	No	3	0.0%	99.8%
Prevotella intermedia	No	3	0.0%	99.8%
Prevotella oralis	No	3	0.0%	99.8%
Prevotella oris	No	3	0.0%	99.8%
Pseudomonas fluorescens	No	3	0.0%	99.8%
Pseudomonas oryzihabitans	No	3	0.0%	99.8%
Psychrobacter sanguinis	No	3	0.0%	99.8%
Rahnella species	No	3	0.0%	99.8%
Ralstonia species	No	3	0.0%	99.8%
Roseomonas species	No	3	0.0%	99.8%
Salmonella kentucky	No	3	0.0%	99.8%
Salmonella napoli	No	3	0.0%	99.8%
Salmonella oranienburg	No	3	0.0%	99.8%
Salmonella rissen	No	3	0.0%	99.8%
Shewanella putrefaciens	No	3	0.0%	99.8%
Staphylococcus auricularis	Yes	3	0.0%	99.8%
Turicella species	No	3	0.0%	99.8%
Vibrio cholerae	No	3	0.0%	99.8%
Vibrio parahaemolyticus	No	3	0.0%	99.8%
Weissella confusa	No	3	0.0%	99.8%
Acinetobacter pittii	No	2	0.0%	99.8%
Aeromonas sobria	No	2	0.0%	99.8%
Alloscardovia omnicolens	No	2	0.0%	99.8%
Anaerobic gramnegative and gram	No	2	0.0%	99.8%
Anaerococcus lactolyticus	No	2	0.0%	99.8%
Anaerococcus octavius	No	2	0.0%	99.8%
Bacteroides nordii	No	2	0.0%	99.8%
Bacteroides salyersiae	No	2	0.0%	99.8%
Bordetella bronchiseptica	No	2	0.0%	99.8%
Brevibacterium ravenspurgense	Yes	2	0.0%	99.8%
Butyricimonas virosa	No	2	0.0%	99.8%
Campylobacter coli	No	2	0.0%	99.8%

Candida pulcherrima	No	2	0.0%	99.8%
Citrobacter farmeri	No	2	0.0%	99.8%
Clostridium butyricum	No	2	0.0%	99.8%
Clostridium fallax	No	2	0.0%	99.8%
Clostridium indolis	No	2	0.0%	99.8%
Corynebacterium coyleae	Yes	2	0.0%	99.8%
Corynebacterium glucuronolyticum	Yes	2	0.0%	99.8%
Corynebacterium jeikeium (jk)	Yes	2	0.0%	99.8%
Corynebacterium urealyticum	Yes	2	0.0%	99.8%
Cupriavidus metallidurans	No	2	0.0%	99.8%
Cutibacterium avidum	Yes	2	0.0%	99.8%
Dermabacter species	Yes	2	0.0%	99.8%
Dysgonomonas species	No	2	0.0%	99.8%
Egerthella species	No	2	0.0%	99.8%
Eggerthia catenaformis	No	2	0.0%	99.8%
Elizabethkingia species	No	2	0.0%	99.8%
Erwinia species	No	2	0.0%	99.8%
Ewingella americana	No	2	0.0%	99.8%
Fungus	No	2	0.0%	99.8%
Globicatella sulfidifaciens	No	2	0.0%	99.8%
Globicatella sulfidofaciens	No	2	0.0%	99.9%
Gordonia terrae	No	2	0.0%	99.9%
Hathewaya limosa	No	2	0.0%	99.9%
Helcococcus kunzii	No	2	0.0%	99.9%
Klebsiella terrigena	No	2	0.0%	99.9%
Kluyvera cryocrescens	No	2	0.0%	99.9%
Kocuria kristinae	Yes	2	0.0%	99.9%
Kocuria varians	Yes	2	0.0%	99.9%
Microaerophilic grampositive cocci	No	2	0.0%	99.9%
Mixed flora	Yes	2	0.0%	99.9%
Myroides species	No	2	0.0%	99.9%
Neisseria sicca	No	2	0.0%	99.9%
Oligella urethralis	No	2	0.0%	99.9%
Paracoccus species	No	2	0.0%	99.9%
Pediococcus acidilactici	No	2	0.0%	99.9%
Peptoniphilus gorbachii	No	2	0.0%	99.9%
Peptostreptococcus tetradius	No	2	0.0%	99.9%
Pseudoclavibacter species	Yes	2	0.0%	99.9%
Pseudomonas monteilii	No	2	0.0%	99.9%
Ralstonia mannitolilytica	No	2	0.0%	99.9%
Raoultella terrigena	No	2	0.0%	99.9%
Salmonella agona	No	2	0.0%	99.9%
Salmonella corvallis	No	2	0.0%	99.9%
Salmonella grumpensis	No	2	0.0%	99.9%
Salmonella infantis	No	2	0.0%	99.9%
Salmonella reading	No	2	0.0%	99.9%
Salmonella sandiego	No	2	0.0%	99.9%
Serratia odorifera	No	2	0.0%	99.9%
Shigella flexneri	No	2	0.0%	99.9%
Sphingobacterium multivorum	No	2	0.0%	99.9%
Staphylococcus lentus	Yes	2	0.0%	99.9%
Staphylococcus xylosus	Yes	2	0.0%	99.9%
Streptococcus infantarius	No	2	0.0%	99.9%
Streptococcus macedonicus	No	2	0.0%	99.9%
Streptococcus uberis	No	2	0.0%	99.9%
Synergistes species	No	2	0.0%	99.9%

Weissella species	No	2	0.0%	99.9%
Abiotrophia adiacens	No	1	0.0%	99.9%
Acidaminocuccus intestini	No	1	0.0%	99.9%
Acidovorax species	No	1	0.0%	99.9%
Acinetobacter radioresistans	No	1	0.0%	99.9%
Actinobaculum urinale	No	1	0.0%	99.9%
Actinomyces cardiffensis	No	1	0.0%	99.9%
Actinomyces graevenitzii	No	1	0.0%	99.9%
Actinotignum massiliense	No	1	0.0%	99.9%
Alcaligenes species	No	1	0.0%	99.9%
Anaerobic growth	No	1	0.0%	99.9%
Anaerobic streptococci	No	1	0.0%	99.9%
Arcobacter butzleri	No	1	0.0%	99.9%
Aspergillus fumigatus	No	1	0.0%	99.9%
Aspergillus species	No	1	0.0%	99.9%
Bacteroides splanchnicus	No	1	0.0%	99.9%
Bergeyella zoohelcum	No	1	0.0%	99.9%
Bifidobacterium dentium	No	1	0.0%	99.9%
Bordetella holmesii	No	1	0.0%	99.9%
Bordetella-art	No	1	0.0%	99.9%
Brevibacterium casei	Yes	1	0.0%	99.9%
Brevibacterium luteolum	Yes	1	0.0%	99.9%
Brevundimonas vesicularis	No	1	0.0%	99.9%
Burkholderia gladioli	No	1	0.0%	99.9%
Burkholderia pseudomallei	No	1	0.0%	99.9%
Campylobacter concisus	No	1	0.0%	99.9%
Campylobacter curvus	No	1	0.0%	99.9%
Campylobacter lari	No	1	0.0%	99.9%
Campylobacter showae	No	1	0.0%	99.9%
Candida lipolytica	No	1	0.0%	99.9%
Cellulosimicrobium cellulans	Yes	1	0.0%	99.9%
Chryseobacterium meningosepticur	No	1	0.0%	99.9%
Clostridium bifermentans	No	1	0.0%	99.9%
Clostridium disporicum	No	1	0.0%	99.9%
Clostridium hathewayi	No	1	0.0%	99.9%
Clostridium limosum	No	1	0.0%	99.9%
Corynebacterium afermentans	Yes	1	0.0%	99.9%
Corynebacterium confusum	Yes	1	0.0%	99.9%
Corynebacterium kroppenstedtii	Yes	1	0.0%	99.9%
Corynebacterium tuberculostearicui	Yes	1	0.0%	99.9%
Corynebacterium ulcerans	Yes	1	0.0%	99.9%
Cryptococcus laurentii	No	1	0.0%	99.9%
Curtobacterium species	No	1	0.0%	99.9%
Delftia species	No	1	0.0%	99.9%
Edwardsiella species	No	1	0.0%	99.9%
Elizabethkingia meningoseptica	No	1	0.0%	99.9%
Elizabethkingia miricola	No	1	0.0%	99.9%
Enterococcus cecorum	No	1	0.0%	99.9%
Enterococcus mundtii	No	1	0.0%	99.9%
Enterococcus thailandicus	No	1	0.0%	99.9%
Facklamia languida	No	1	0.0%	99.9%
Facklamia sourekii	No	1	0.0%	99.9%
Filifactor species	No	1	0.0%	99.9%
Flavonifractor species	No	1	0.0%	99.9%
Fusobacterium naviforme	No	1	0.0%	99.9%
Fusobacterium periodonticum	No	1	0.0%	99.9%

Fusobacterium varium	No	1	0.0%	100.0%
Gordonibacter pamelaeae	No	1	0.0%	100.0%
Grampositive mixed flora	Yes	1	0.0%	100.0%
Haemophilus haemolyticus	No	1	0.0%	100.0%
Haemophilus parahaemolyticus	No	1	0.0%	100.0%
Haemophilus species	No	1	0.0%	100.0%
Helcobacillus massiliensis	No	1	0.0%	100.0%
Klebsiella ornithinolytica	No	1	0.0%	100.0%
Lachnoanaerobaculum species	No	1	0.0%	100.0%
Lactobacillus catenaformis	Yes	1	0.0%	100.0%
Lactobacillus fermentum	Yes	1	0.0%	100.0%
Listeria innocua	No	1	0.0%	100.0%
Microaerophilic grampositive rods	No	1	0.0%	100.0%
Moraxella lacunata	No	1	0.0%	100.0%
Neisseria bacilliformis	No	1	0.0%	100.0%
Neisseria gonorrhoeae	No	1	0.0%	100.0%
Ochrobactrum intermedium	No	1	0.0%	100.0%
Paenibacillus amylolyticus	No	1	0.0%	100.0%
Paraclostridium bifermentans	No	1	0.0%	100.0%
Peptoniphilus lacrimalis	No	1	0.0%	100.0%
Peptostreptococcus anaerobius	No	1	0.0%	100.0%
Prevotella buccalis	No	1	0.0%	100.0%
Prevotella disiens	No	1	0.0%	100.0%
Proteus penneri	No	1	0.0%	100.0%
Pseudoglutamicibacter species	Yes	1	0.0%	100.0%
Remove	No	1	0.0%	100.0%
Rhodococcus corynebacterioides	No	1	0.0%	100.0%
Rothia aeria	Yes	1	0.0%	100.0%
Salmonella agbeni	No	1	0.0%	100.0%
Salmonella altona	No	1	0.0%	100.0%
Salmonella amager	No	1	0.0%	100.0%
Salmonella anatum	No	1	0.0%	100.0%
Salmonella bareilly	No	1	0.0%	100.0%
Salmonella bovis-morbificans	No	1	0.0%	100.0%
Salmonella bredeney	No	1	0.0%	100.0%
Salmonella colindale	No	1	0.0%	100.0%
Salmonella durban	No	1	0.0%	100.0%
Salmonella florida	No	1	0.0%	100.0%
Salmonella gaminara	No	1	0.0%	100.0%
Salmonella give	No	1	0.0%	100.0%
Salmonella grupp o 13	No	1	0.0%	100.0%
Salmonella grupp o 2	No	1	0.0%	100.0%
Salmonella grupp o4	No	1	0.0%	100.0%
Salmonella hadar	No	1	0.0%	100.0%
Salmonella havana	No	1	0.0%	100.0%
Salmonella javiana	No	1	0.0%	100.0%
Salmonella kottbus	No	1	0.0%	100.0%
Salmonella livingstone	No	1	0.0%	100.0%
Salmonella london	No	1	0.0%	100.0%
Salmonella richmond	No	1	0.0%	100.0%
Salmonella senftenberg	No	1	0.0%	100.0%
Salmonella stanleyville	No	1	0.0%	100.0%
Salmonella weltevreden	No	1	0.0%	100.0%
Salmonella wien	No	1	0.0%	100.0%
Selenomonas species	No	1	0.0%	100.0%
Sneathia sanguinegens	No	1	0.0%	100.0%

Sporobolomyces salmonicolor	No	1	0.0%	100.0%
Sporosarcina species	No	1	0.0%	100.0%
Staphylococcus equorum	Yes	1	0.0%	100.0%
Stenotrophomonas species	No	1	0.0%	100.0%
Streptococcus infantis	No	1	0.0%	100.0%
Streptococcus ovis	No	1	0.0%	100.0%
Streptokockart	No	1	0.0%	100.0%
Tsukamurella species	No	1	0.0%	100.0%
Veillonella atypica	No	1	0.0%	100.0%
